# Serotonergic, Dopaminergic, and Noradrenergic Modulation of Erotic Stimulus Processing in the Male Human Brain

**DOI:** 10.3390/jcm8030363

**Published:** 2019-03-14

**Authors:** Heiko Graf, Kathrin Malejko, Coraline Danielle Metzger, Martin Walter, Georg Grön, Birgit Abler

**Affiliations:** 1 Department of Psychiatry and Psychotherapy III, Ulm University, 89075 Ulm, Germany; kathrin.malejko@uni-ulm.de (K.M.); georg.groen@uni-ulm.de (G.G.); birgit.abler@uni-ulm.de (B.A.); 2 Department of Psychiatry, Otto von Guericke University, 39120 Magdeburg, Germany; coraline.metzger@med.ovgu.de; 3 Institute of Cognitive Neurology and Dementia Research (IKND), Otto von Guericke University, 39106 Magdeburg, Germany; 4 German Center for Neurodegenerative Diseases (DZNE), 39120 Magdeburg, Germany; 5 Department of Psychiatry, Eberhard Karls University, 72074 Tuebingen, Germany; martin.walter@uni-tuebingen.de; 6 Leibniz Institute for Neurobiology, 39120 Magdeburg, Germany

**Keywords:** erotic stimulus processing, serotonin, noradrenaline, dopamine, fMRI, healthy, human

## Abstract

Human sexual behavior is mediated by a complex interplay of cerebral and spinal centers, as well as hormonal, peripheral, and autonomic functions. Neuroimaging studies identified central neural signatures of human sexual responses comprising neural emotional, motivational, autonomic, and cognitive components. However, empirical evidence regarding the neuromodulation of these neural signatures of human sexual responses was scarce for decades. Pharmacological functional magnetic resonance imaging (fMRI) provides a valuable tool to examine the interaction between neuromodulator systems and functional network anatomy relevant for human sexual behavior. In addition, this approach enables the examination of potential neural mechanisms regarding treatment-related sexual dysfunction under psychopharmacological agents. In this article, we introduce common neurobiological concepts regarding cerebral sexual responses based on neuroimaging findings and we discuss challenges and findings regarding investigating the neuromodulation of neural sexual stimulus processing. In particular, we summarize findings from our research program investigating how neural correlates of sexual stimulus processing are modulated by serotonergic, dopaminergic, and noradrenergic antidepressant medication in healthy males.

## 1. Introduction

Human sexual behavior is mediated by the integration of endocrine, vascular, peripheral, and central nervous mechanisms. The brain is considered as the “master organ” of sexual functioning [1] and is involved in all successive steps of human sexual behavior [2]. Electrophysiological and behavioral studies provided considerable insights into human sexual function, but underlying neural substrates were largely unknown until functional neuroimaging methods were widely introduced into neuroscientific research. Since then, the basic principles of neural processing of sexual stimulation were described in several studies [2,3,4]. However, empirical evidence regarding the effects of neuromodulators on these neural mediators was scarce. 

Pharmacological functional magnetic resonance imaging (pharmaco-fMRI) provides a valuable tool to examine modulatory effects of different neurotransmitter systems on neural signatures of sexual function. Apart from clarifying these basic principles and the complex interaction between neuromodulators and functional network anatomy of sexual behavior, investigations with pharmaco-fMRI also have the potential to elucidate the neural correlates of treatment-related sexual dysfunction. 

Various psychiatric disorders are commonly accompanied by sexual dysfunction, and have an immediate impact on subjective well-being and quality of life [5,6]. Of note, sexual dysfunction also occurs as a frequent side effect of psychopharmacological treatment and considerably compromises adherence to therapy. Clinical observational studies suggest that about 40% of patients with psychopharmacological antidepressant treatment discontinue their medication due to treatment-related sexual dysfunction [7]. Thus, the crucial implication of sexual dysfunction as a disease- and treatment-related symptom motivated the investigation of the underlying neural mechanisms.

In this review, we introduce common concepts of sexual behavior and evidence regarding neural substrates of sexual responses. We shortly discuss the challenges investigating the neuromodulation of neural sexual stimulus processing by pharmaco-fMRI. In particular, we summarize our research program that focused on how these neural correlates were modulated by serotonergic, dopaminergic, and noradrenergic antidepressant medication in healthy male subjects.

## 2. Conceptualizing Sexual Behavior and Neural Responses

Despite its debut already in the 1960s, the most commonly used model to conceptualize sexual activity is still the sexual response cycle by Masters and Johnson [8]. The term “sexual response” denotes the set of behaviors and functions related to sexual stimulation and the pursuit of a sexual goal. Based on their observations, Masters and Johnson [8] defined four different phases of sexual responses that refer to the sequence of physical and emotional changes during sexual arousal and activity. They distinguished a period of sexual desire and arousal, followed by a plateau, culminating in orgasm and ending in a refraction period. Kaplan proposed a slightly modified triphasic model comprising sexual desire, excitement, and orgasm [9]. However, these models were criticized for the linear sequence of the phases that may, for example, not be entirely transferable to female sexual responses. 

Neuroimaging techniques such as positron emission tomography (PET) or functional magnetic resonance imaging (fMRI) made valuable contributions to identify underlying neural correlates of sexual responses. As outlined and summarized by Reference [10], the behavioral and neurofunctional principles underlying the sexual response cycle largely overlap with those related to other primary rewards such as food [10]. Analogous to concepts related to other rewards, Georgiadis and Kringelbach [10] suggested that sexual responses may be characterized by terms of motivation–consummation–satiety or wanting–liking–inhibition. Linking psychological with physiological and neurofunctional processes in more detail, a meta-analysis of functional imaging studies on sexual arousal conceptualized the neurophenomenological model of sexual arousal [2]. The model suggests a cognitive component, comprising the appraisal of and the attention to a subsequent sexual stimulus, which is represented by neural reactivity within the orbitofrontal cortex (OFC), the inferior temporal cortices, the inferior and superior parietal lobules, premotor and supplementary motor areas, and within the cerebellum. An emotional component representing sexual pleasure and hedonic qualities of sexual arousal as a primary reward is suggested to be mediated by neural activations of the amygdala, the insula, and primary and secondary somatosensory cortices. Neural processes comprising goal-directed behavior and the perceived urge to express overt sexual behavior are represented by activations within the anterior cingulate cortex (ACC), the claustrum, the posterior parietal cortex, the hypothalamus, the substantia nigra, and the ventral striatum. The autonomic/neuroendocrine component is thought to be mediated by activations within the ACC, the anterior insula, the putamen, and hypothalamus, and is supposed to lead subjects to a state of physiological readiness for sexual behavior [2,3,11]. 

A more recent meta-analysis [12] distinguished brain networks underlying psychosexual and physiosexual arousal. Hereby, the psychosexual network was suggested to include the lateral prefrontal cortex and the hippocampus (cognitive and memory-guided evaluations), the occipitotemporal cortex, superior parietal lobules (sensory processing), the amygdala and the thalamus (relevance detection and affective evaluation), the hypothalamus (autonomic responses), basal ganglia (sexual urge), and the anterior insula (awareness of sexual arousal). Physiosexual processes were conceptualized within a network comprising the subgenual anterior cingulate cortex (sgACC; autonomic and corresponding emotion regulation), the anterior midcingulate cortex (aMCC; initiation of copulatory behavior), the putamen and claustrum (sexual urge), the anterior insula (awareness of rising sexual desire and engendered bodily reactions), the insular cortex (somatosensory information), and the operculum (monitoring bodily changes during sexual arousal). Of note, the putamen and the claustrum were identified as brain regions that connect both psychosexual und physiosexual networks, with potentially dissociable functions. While the putamen is thought to orchestrate the integration of sensorimotor information in the context of sexual desire, the putamen might be responsible for cross-modal processing between and within the networks of sexual arousal. 

Most of these studies summarized neural sexual responses that were investigated using visual stimuli. However, slightly divergent patterns of brain activations were reported due to different stimulus content (e.g., sexual intensity), presentation mode (visual static images versus dynamic video sequences), or design type (block versus event-related design) [13]. While sexual motivation and wanting is reliably induced by visual sexual stimulation, genital stimulation is usually required to enter the consummatory plateau [4] of the sexual response cycle. Indeed, the use of other types of stimulus material (e.g., haptic or acoustic) is limited by the circumstances of neuroimaging methods, like noise and motion sensitivity. A few studies simultaneously recorded fMRI blood oxygenation level dependent (BOLD) signals elicited by visual stimuli and the corresponding time course of penile tumescence to investigate neural substrates of orgasm and erection. Neural activations within the ACC, the insula, amygdala, hypothalamus, and secondary somatosensory cortices were considered to be associated with penile erection [14,15]. Neural activations in mid-anterior and medial subregions of the OFC were suggested to relate specifically to orgasm [16]. Only few neuroimaging studies investigated sexual inhibition/refraction; however, these were mainly in subjects with low sexual desire. These studies indicate that sexual inhibition is mediated by prefrontal hyperactivity [17,18]. Accordingly, volitional inhibition of sexual arousal in healthy subjects was indeed accompanied by increased activations within the superior parietal, the ventrolateral prefrontal [19], and the inferior frontal cortex [20]. Moreover, it was suggested that both intended and unintended sexual inhibition are related to an exaggerated activity within the neural network of sexual interest that may, however, prevent a shift to the neural sexual consummation network [4]. Investigating neural responses a few minutes after ejaculation, one fMRI study linked activation of the amygdala, the temporal lobes, and the septal area specifically to sexual satiety [21]. 

## 3. Neuromodulation of Sexual Responses

Despite these valuable insights arising from neuroimaging studies into potentially underlying neural correlates of sexual responses, modulatory effects of neurotransmitter systems or monoaminergic drugs like antidepressants on these neural substrates are largely unknown. Most of the evidence regarding the neuromodulation of sexual functions stems from animal studies (e.g., References [22,23]) or clinical observations in patients during the treatment with psychoactive drugs (e.g., References [7,24,25,26]). Understanding the underlying mechanisms is indeed of great relevance considering the high prevalence of psychopharmacologically related sexual dysfunction, quite likely arising from central nervous rather than peripheral mechanisms [27]. 

Apart from sexual hormones and neuropeptides, central monoamines and catecholamines that are commonly modulated by psychopharmacological agents exert a pivotal role in the neuromodulation of sexual behavior. Here, we concentrate on dopamine, serotonin, and noradrenaline as the most commonly altered neuromodulator systems in psychopharmacotherapy. While an elevated central dopaminergic neurotransmission was observed to be accompanied by increased sexual interest, serotonergic agents are associated with an opposite pattern of behavior [27,28,29]. Considering the overlap of behavioral and neurofunctional principles of sexual functioning with other primary rewards [10], the favorable effects of dopamine on sexual behavior seem plausible. Accordingly, the antidepressant and selective noradrenaline and dopamine reuptake inhibitor (SSNDRI) bupropion is associated with subjectively improved sexual functioning, such as the ability to achieve and maintain an erection and orgasm, along with increased sexual satisfaction [30]. Moreover, dopamine-agonist treatment in Parkinson’s disease is frequently accompanied by the clinical observation of hypersexuality [31]. In contrast, up to 70% of patients with schizophrenia report sexual dysfunction under treatment with antidopaminergic antipsychotics like haloperidol [32]. Apart from hyperprolactinemia due to dopamine D_2_-receptor blockage in the tuberoinfundibular pathway [33], the inhibitory effects of dopamine antagonists on the mesolimbic/mesocortical reward system are considered as a crucial mechanism underlying antipsychotic related sexual dysfunction [34]. 

The considerable impact of the neuromodulator serotonin in mediating sexual activity was recognized by the rising prevalence of sexual dysfunction during antidepressant medication, in particular with selective serotonin reuptake inhibitors (SSRIs) [35]. Although the stimulation of some specific serotonin receptor subtypes, e.g., 5-HT_2c_- or 5-HT_1A_-receptors, may facilitate erection or ejaculation, primary central serotonergic effects are thought to be inhibitory. These effects are presumably mediated via decreased dopamine release in mesolimbic regions [28,36] and by suppressing spinal ejaculatory centers [37]. Accordingly, up to 80% of patients treated with the SSRI sertraline report sexual dysfunction and, in particular in young patients, antidepressant-related decrease in sexual function is one of the most relevant side effects [24,38]. Apart from the immediate negative impact on the quality of life [39,40], antidepressant-related sexual dysfunction is also one of the major reasons that lead to non-adherence to treatment [41], especially after remission of depressive symptoms. Since early discontinuation compared to the recommended maintenance therapy over several months is related to increased rates of relapse [42], the side effect compromises the overall success of antidepressant treatment. 

Compared to serotonin, the contribution of the neuromodulator noradrenaline in mediating sexual responses is less well understood. Clinical observations assume a favorable effect of selective noradrenaline reuptake inhibitors (SNRIs) on sexual functions compared to SSRIs based on lower rates of sexual dysfunction under SNRIs [24,43,44]. In line with this, actual sexual activity is related with an increase in plasma noradrenaline levels during orgasm with a subsequent rapid decline [45]. However, the limited available data regarding the effects of SNRIs on sexual functioning compromise definite conclusions [24]. 

## 4. Challenges

The conclusions regarding the effects of monoaminergic psychopharmaceuticals on sexual functions are mainly based on clinical observations and may be confounded by the disease itself. Most studies did not assess baseline sexual function before the initiation of medication. However, up to 75% of patients with major depression report sexual dysfunction prior to antidepressant treatment, in particular decreased sexual interest [46,47]. Thus, the mechanisms related to sexual dysfunction under monoaminergic agents have also to be investigated in healthy subjects to exclude confounds by the disease itself. Moreover, to meet clinical conditions as much as possible, but also to reach steady-state conditions, multi-dose trials over several days rather than single-dose applications are required to investigate neural correlates of sexual responses under antidepressants. Another limitation often arises from the study design, especially when two agents are compared with each other or relative to placebo in two different study groups. These study designs limit the capability to differentiate effects of group from those of medication, even when randomization was applied to minimize between-group effects. Also, between-group designs usually require larger sample sizes to reduce putative and systematic effects of group. Thus, apart from placebo-controlled investigations in healthy subjects under subchronic administration of study medication, repeated measures within one group (within-subject and cross-over) may represent the most desirable study design to investigate psychopharmacological effects on neural responses of sexual behavior. 

## 5. Serotonergic, Dopaminergic, and Noradrenergic Neuromodulation of Sexual Responses

One of the first studies investigating sexual dysfunction under monoaminergic agents and underlying neural correlates was conducted in 2009 [48] in male patients with major depression. Neural activations under visual erotic stimulation in nine patients taking SSRIs (six took paroxetine and three fluoxetine) and in 10 patients taking mirtazapine, which blocks central adrenergic and serotonin receptors, were compared to 10 healthy controls. This study demonstrated decreased neural activation within the ACC, the OFC, the insula, and the caudate nucleus in the SSRI-group compared to controls. These brain regions with attenuated responses were related to attentional and motivational components of the sexual response cycle. Neural activations in the group treated with mirtazapine were relatively lower than in controls but still elevated compared to those treated with SSRIs. Sexual dysfunction as assessed by questionnaires was significantly more frequent in depressed patients compared to controls, but did not differ between the two treatment groups. This study provided first evidence for the potential underlying neural correlates of sexual dysfunction in depression while under antidepressant treatment. However, the study design was not in the position to distinguish effects of disease from treatment-related effects on sexual functions. 

We, therefore, investigated a sample of 18 healthy heterosexual males using fMRI and a randomized placebo-controlled within-subject cross-over study design. Participants were investigated after subchronic administration of the SSRI paroxetine, the SSNDRI bupropion, and placebo. Each treatment was applied for seven days separated by a wash-out time of at least 14 days [49]. During fMRI, we used a dynamic visual erotic stimulus paradigm consisting of erotic and non-erotic video clips. Erotic video clips depicted sexual interactions between one man or two women (petting, oral sex, and vaginal intercourse) extracted from commercial adult films. Non-erotic video clips showed men and women in emotionally neutral interactions. Subjective behavioral changes in sexual interest, sexual arousal, the ability to achieve orgasm, the ability to achieve and maintain an erection, and overall sexual satisfaction during drug administration were assessed by the Massachusetts General Hospital Sexual Functioning Questionnaire (MGH-SFQ) [50]. We demonstrated significantly attenuated neural activations within the sgACC, the pgACC, the aMCC, the pMCC, the nucleus accumbens, the midbrain, and the amygdala under the SSRI during visual erotic stimulation. In line with these neural alterations under the SSRI, we found a decrease in subjective sexual functions under paroxetine compared to placebo. In particular, we observed a significant decrease in subjective sexual arousal and the ability to achieve an orgasm under the SSRI compared to placebo. 

Neural activations within the anterior but also rather rostral subdivisions of the ACC were previously found to be modulated by SSRIs during emotional aversive stimuli [51]. Within the context of sexual behaviour, neural activations within the ACC are associated with autonomic components of sexual responses [2,3]. Moreover, neural activity within the pgACC is related to the interaction of subjective sexual intensity and its hedonic and emotional value [52]. The results, therefore, suggested an altered neural reactivity within brain regions linked to autonomic and emotional components of sexual responses under SSRIs. In particular, attenuated neural activations within the pMCC under the SSRI were correlated with paroxetine blood-serum levels and with detrimental overall subjective functions under this antidepressant. In addition, by demonstrating attenuated neural activations within the nucleus accumbens under the SSRI, we found evidence for a diminished neural motivational component of sexual responses. This attenuation may relate to the close interaction and opposing effects between dopaminergic and serotonergic systems [53,54,55]. Increasing levels of serotonin as seen under SSRIs seem to dampen the functioning of the dopaminergic reward system [54,56]. To further examine whether the SSRI-related attenuation of the dopaminergic reward system and, in particular, within the nucleus accumbens might be mediated by other brain regions as observed in secondary rewards [57], we applied a psychophysiological interaction approach [58]. Indeed, we observed a significantly elevated negative reciprocal interaction between the anteroventral prefrontal cortex (avPFC) and the nucleus accumbens under the SSRI that was also associated with impulsivity as a personality trait. Thus, an increase in PFC activation may mediate the dampening effects of SSRIs on the human reward system and associated functions, e.g., sexual satisfaction. 

**Figure 1:**
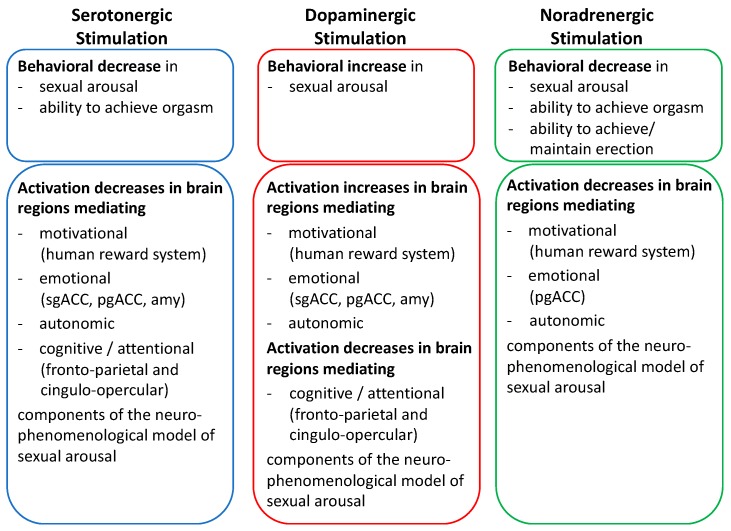
Implications of subchronic steady state serotonergic, noradrenergic, and dopaminergic stimulation on subjective sexual functions and neural responses to erotic stimulation in healthy subjects. For further information on the neurophenomenological model of sexual arousal, see Stoléru et al. [2]. SgACC = subgenual anterior cingulate cortex, pgACC = pregenual anterior cingulate cortex, amy = amygdala.

In line with the opposing effects of serotonin and dopamine on reward-related functions and neural activity, we observed slightly enhanced and prolonged neural activations within the pMCC and within subcortical regions such as the midbrain, the amygdala, and the thalamus under the SSNDRI bupropion compared to placebo. Subjective sexual functions were indeed unimpaired under this agent in accordance to clinical studies, suggesting bupropion as a treatment alternative in patients with SSRI-related sexual dysfunction [59]. The dopaminergic agents also reveal favourable effects on sexual functions as compared to SSRIs [59,60,61,62]. The elevated neural activation pattern as found in our study and, in particular, within the ventral striatum and the midbrain as dopaminergic reward-related brain regions may represent a neural correlate of increased responsiveness to sexual stimuli arising from the dopaminergic properties of bupropion. Moreover, with concomitant activations within the amygdala that were previously related to perceived sexual arousal and to orgasmic pleasure [63], and neural activations within the thalamus and cortical regions such as the MCC, we observed activations within a neural network referred to as the salience network, which integrates homeostatic autonomic functions, emotion, and reward processing [64] (see Figure 1).

Apart from these diverging effects of the neuromodulators serotonin and dopamine, a unidirectional neural activation was found under both the SSRI and the SSNDRI within the aMCC and, thus, in a brain region associated with attentional top-down control [65]. However, the video-clip task limited the specific investigation of attentional components of sexual responses. We, therefore, investigated the same sample of 18 healthy male subjects with fMRI under the two antidepressants paroxetine and bupropion compared to placebo. During fMRI, we now used an established visual erotic picture task [52,66] consisting of erotic and non-erotic pictures of positive emotional content taken from the International Affective Picture System (IAPS) [67]. Of note, half of the stimuli of each condition (erotic, non-erotic) were announced. The implementation of these anticipatory periods allowed the reliable investigation of attentional processes [68]. In general, anticipation is regarded as preceding attention to an upcoming predicted stimulus [69,70] and numerous studies showed neural parallels between anticipatory and attentional processes [68,71]. Under both serotonergic and dopaminergic antidepressants, we revealed attenuated neural activations within the fronto-parietal and cingulo-opercular neural network, essential for task initiation and adjustment, as well as for the maintenance of attention [65]. Accordingly, these network alterations were accompanied by unidirectional detrimental effects on the behavioral level under both agents in terms of prolonged reactions in a divided attention task. 

Beneficial effects of increasing dopaminergic neurotransmission on attention and prefrontal cortical functions were conceptualized as an inverted u-shaped curve [72], whereby either too low or too high levels of dopamine [73] led to a worsening of prefrontal cortex functioning. Thus, one may argue that an increase in dopaminergic neurotransmission in healthy subjects as induced by the SSNDRI bupropion may have increased the responsivity of the neural attention network beyond the optimum and led to detrimental attentional functioning on a behavioral level. A similar response pattern was shown for increases in noradrenergic neurotransmission and other cognitive functions such as error monitoring [74]. In addition, it is of note that an increase in dopaminergic neurotransmission in prefrontal regions is not only described for bupropion but also for paroxetine via indirect pathways [75,76], supporting our observation regarding similar attention network alterations. In line with this, detrimental sustained attention was also found in other studies under SSRI administration in healthy subjects [77,78]. 

Apart from the serotonergic and dopaminergic antidepressants, we further investigated neural effects of noradrenergic antidepressants. Within a randomized placebo-controlled within-subject cross-over design, 19 healthy heterosexual male subjects were investigated after subchronic administration of the selective noradrenaline reuptake inhibitor (SNRI) reboxetine and the second-generation antipsychotic amisulpride. During fMRI, we again used the dynamic erotic video-clip task. Noradrenergic agents and, in particular, reboxetine were thought to exert less detrimental effects on sexual functioning compared to serotonergic agents [24,79]. However, this assumption was mainly derived from investigations in depressive patients that demonstrated greater improvement in sexual satisfaction, in the ability to become sexually excited [80], and in achieving orgasm [43] under reboxetine. In contrast to these beneficial effects on sexual functions, we observed a significant decrease in overall subjective sexual function under the noradrenergic agent reboxetine compared to placebo and amisulpride in healthy subjects. In particular sexual arousal, the ability to achieve orgasm and penile erection [81] decreased. These results were, however, in line with other previous clinical reports of prolonged orgasm [82], erectile dysfunction [83], and anorgasmia [43] under this drug. On the neural level, we revealed diminished neural activations within the caudate nucleus under reboxetine compared to placebo that were significantly associated with the decreased sexual interest under this agent. With regard to erotic stimulation, caudate nucleus activation was linked to goal-directed behavior and reward [84]. Whereas ventral parts of the striatum are commonly associated with the expectation and the receipt of incentives, dorsal striatal/caudate nucleus activation was associated with motivational rather than reward processing [85]. Thus, our findings may support the notion that an increase in noradrenergic neurotransmission might have detrimental effects on motivational components of sexual responses along with diminished subjective sexual functioning. 

It is of note that we did not find significant neural alterations during visual erotic stimulation and in subjective sexual functions under the antipsychotic drug amisulpride compared to placebo. The antipsychotic drug amisulpride has high and selective affinity to postsynaptic D_2_- und D_3_-receptors [86,87,88] and it is known for its capacity to induce sexual dysfunction mainly due to the blockage of dopamine D_2_-receptors [33] with secondary increases of prolactin levels [34]. The lack of significant alterations in neural visual erotic stimulus processing along with unchanged subjective sexual functions in our study was most likely due to the low dosage of 200 mg/day amisulpride for seven days. Antipsychotic effects of amisulpride were reported for high dosages from about 400 to 600 mg/day due to reliable D_2_-receptor occupancy [86]. In contrast, lower dosages (50 to 200 mg/day) as used in our study are thought to primarily block presynaptic dopamine autoreceptors with the consequence of mild pro-dopaminergic effects [86,89,90] that may have left sexual functions and corresponding neural correlates unimpaired in our sample of healthy male subjects. 

To further investigate neural responses to visual erotic stimulus processing including preceding attention and their modulation by noradrenergic agents, we also applied the abovementioned erotic picture paradigm with anticipatory periods [91]. Notably, upon static rather than previously applied dynamic visual erotic stimulation, we observed additional treatment effects of the noradrenergic agent reboxetine compared to placebo during visual erotic stimulation by diminished neural activations not only within the caudate nucleus, but also within the ventral striatum/nucleus accumbens, the pgACC, the aMCC, and the OFC. In addition, decreases in subjective sexual arousal correlated with attenuated neural activations within the posterior insula, a region that is repeatedly associated with sexual arousal and penile response [14,84]. Thus, our results support the notion of detrimental effects of noradrenergic agents on emotional, motivational, and autonomic neural components of sexual responses, along with decreased subjective sexual function (see Figure 1). In addition, they also underpin the implication regarding stimulus presentation mode in investigating neural substrates of erotic stimulus processing considering that treatment effects of noradrenergic agents were found within a broader neural network during static rather than dynamic visual erotic stimulation. 

Similar to the investigation by erotic video stimulation, we also found no significant neural alteration under amisulpride compared to placebo. Moreover, in contrast to serotonergic and predominantly dopaminergic antidepressants, neither the noradrenergic agent reboxetine nor the antipsychotic amisulpride led to neural alterations during the anticipation of erotic stimuli, in line with unimpaired attentional functions on a behavioral level in this study. However, it is of note that major nodes of the neural network altered by the noradrenergic agent reboxetine compared to placebo such as the ventral striatum, the pgACC, aMCC, and the OFC highly resemble those brain regions that were also modulated by serotonergic agents upon erotic video stimulation in our previous investigation. 

While it remains speculative, one may argue that either monoaminergic modulation ends up via similar neural pathways and presumably also on a molecular level. Interactions of both the serotonergic and noradrenergic system with dopaminergic projections were extensively studied [54,92], and a modulation of one system will invariably influence the transmission of the other. Here, the human reward system may represent a major or common final pathway. The specific increase in serotonergic and noradrenergic turnover under paroxetine and reboxetine, respectively, dampened the neural activity within the dopaminergic human reward system and, in particular, within the nucleus accumbens. However, this attenuation is potentially restricted to the processing of specific rewards or reinforcers such as sexual stimuli or primary rewards, considering that the serotonergic and noradrenergic attenuation of neural activity within the nucleus accumbens was not evident when processing monetary rewards as secondary reinforcers [93,94]. 

## 6. Perspectives

Our project using pharmacological and task-based fMRI identified neuromodulatory effects of monoamines and catecholamines on neural sexual responses and potential neural proxies for the development of sexual dysfunction under antidepressants. Insights from this methodological approach mainly concern basic research; however, some aspects might be transferred to clinical practices. Considering that task-based fMRI may not be easily implemented in clinical routines due to its complexity and dependency on a subject’s motivation and performance, resting-state fMRI may provide a valuable alternative. Accordingly, we investigated healthy subjects using resting-state fMRI [95] and demonstrated that more impaired subjective sexual function under serotonergic agents was predicted by low baseline functional connectivities under placebo. In particular, functional connectivities of the sublenticular extended amygdala with midbrain, pgACC, and the insula revealed a predictive potential for the development of SSRI-related decreases in sexual functioning. Although these results await empirical replication in larger samples, they may support the idea of a potentially valuable contribution of imaging techniques in the prediction of pharmaco-related sexual dysfunction within the context of personalized medicine. 

It is of note that the investigations presented were exclusively conducted in healthy male subjects and the conclusions drawn may not be transferable to females. Within the past years, gender and sex aspects were widely recognized in scientific research and, with regard to sexual responses, sex differences are proposed to not only occur on behavioral and downstream peripheral, but also on the neural level. Relative to men, meta-analyses suggest a less consistent and decreased neurofunctional activation in subcortical regions in women during sexual arousal [96,97]. In addition, female sex hormones appear to play a crucial role in mediating particularly cortical activations in response to sexual stimulation [97,98,99]. Moreover, there is evidence for an interaction between sex hormones and the dominant neurotransmitters such as serotonin and dopamine [100]. These observations suggest a divergent monoaminergic neuromodulation of erotic stimulus processing in females. Consequently, the investigation of females under different levels of monoaminergic or catecholaminergic neurotransmitter levels in combination with different hormonal states is highly encouraged as a future research topic.

## 7. Conclusions

Within a broader research program, we investigated healthy male subjects under visual erotic stimulation by fMRI and different antidepressant medication to disentangle effects of monoaminergic and catecholaminergic neuromodulatory substances on neural substrates of sexual responses. After increasing serotonergic neurotransmission, we observed attenuated neural activations within cerebral networks previously related to motivational, emotional, and autonomic components of sexual behavior along with diminished subjective sexual functions. Psychophysiological interaction analyses revealed that the dampening of the motivational component and, in particular, human reward system activation was presumably mediated by an increase in prefrontal cortex activation as a potential correlate of increased cognitive control under serotonergic agents. Of note, neural motivational and emotional components, as well as subjective sexual functions, were either unaffected or even increased under dopaminergic stimulation. Apart from these divergent effects on erotic stimulus processing, both serotonergic and dopaminergic stimulation diminished neural attention network activation during the anticipation of visual sexual stimuli, along with a decrease in behavioral measures of attention. Investigating the noradrenergic neuromodulation of neural substrates of erotic stimulus processing revealed similar neural alterations as serotonergic agents, and showed again attenuation of neural emotional and motivation components along with a decrease in subjective sexual functions. However, neural activations during the anticipation of sexual stimuli and behavioral attentional functioning were not altered by a noradrenergic agent. Thus, our results provided evidence for the neuromodulatory effects of serotonergic, noradrenergic, and dopaminergic agents on neural substrates of erotic stimulus processing. Considering the overlay of neuromodulatory effects of serotonergic and noradrenergic neurotransmission, this may suggest that both monoaminergic modulations end up via similar neural pathways and presumably affect dopaminergic projections within the human reward system. Notably, the dampening of the human reward system by both serotonergic and noradrenergic agents was, however, restricted to the processing of visual sexual stimuli as primary reinforcers and was not evident during processing of monetary rewards as secondary reinforcers. 

From a basic research perspective, we demonstrate that modulations in sexual functioning on the subjective behavioral level are indeed closely linked to cerebral networks that mediate motivational, emotional, autonomic, and attentional components of the sexual response. Our data emphasize the hypothesis that altered cerebral reactivity rather than peripheral effects might be the key to explain side effects of monoaminergic substances on sexual functioning.
